# Effect of extruded adzuki bean flour on the quality and α‐glucosidase inhibitory activity of Chinese steamed bread

**DOI:** 10.1002/fsn3.1181

**Published:** 2019-08-22

**Authors:** Yinhuan Chen, Xiushi Yang, Huimin Guo, Jincai Li, Guixing Ren

**Affiliations:** ^1^ School of Chemical Engineering and Technology Tianjin University Tianjin China; ^2^ Institute of Crop Sciences Chinese Academy of Agricultural Sciences Beijing China

**Keywords:** Chinese steamed bread, extruded adzuki bean flour, nutritional property, sensory evaluation, wheat flour, α‐glucosidase inhibitory activity

## Abstract

In order to improve the commercial and nutritional quality of Chinese steamed bread (CSB), effect of extruded adzuki bean flour (EABF) substitution (10%, 20%, 30%, and 40%) for wheat flour on the quality properties of blended flour and CSB was studied. Results showed that the development time, stability time, and farinograph quality number of blended flours were decreased with EABF addition, although water absorption and softening degree were increased. Pasting properties of them were also degraded with the addition. EABF substitution lowered the lightness and strengthened the hardness of CSB. The sensory evaluation total scores of CSB were both improved with 10% and 20% EABF addition, because slight red color of them was favored by the panelist. Nutritional analysis showed that CSB with 10% and 20% EABF ranked higher protein and essential amino acids content than that of WF‐CSB, especially lysine and threonine. Addition of EABF contributed to the superior α‐glucosidase inhibitory activity of protein isolated from CSBs. α‐Glucosidase inhibition rate of CSB made from EABF (39.88%) was improved nearly five times compared with that of WF. It might be concluded that CSB with addition of 10% and 20% EABF could be used as a nutritional and healthy staple food.

## 1 INTRODUCTION

Chinese steamed bread (CSB), called “Mantou” in Chinese, is a main staple food made from wheat flour and leavening agent. It has been widely consumed in China for thousands of years and is becoming popular in other Asia countries (Wu et al., 2018). CSB could provide carbohydrate and protein to fulfill daily calorie demands for people (Zhu, 2014). It is generally lack of some nutritional components, such as lysine, as well as biological activities (Liu, Mu, Sun, & Chen, 2016). Nowadays, nutritional and biological demands for staple food are increasing to satisfy the interest of health conscious people diet. In recent years, nutrition enrichment of CSB has been studied on the addition of other cereals, to improve antioxidant activity and amino acid balance, such as sorghum and barley (Hao & Beta, 2012; Wu et al., 2018). It is meaningful to supply consumers with nutrition‐balanced and bioactivity‐enhanced CSB, without decreasing its sensory properties.

Adzuki bean (*Vigna angularis*) is a traditional edible legume in China, which contains abundant protein and well‐balanced amino acids. Especially the lysine content in adzuki bean is higher than the FAO/WHO recommended level, which is the first limiting amino acid for most cereals (Tjahjadi, Lin, & Breene, 1988). Moreover, adzuki bean has been suggested as an alternative food for patients with diabetes due to its high resistant starch and phenolic components (Yao, Cheng, Wang, & Ren, 2011). Recently, our research found that extrusion could significantly improve the α‐glucosidase inhibitory activity of adzuki bean on diabetic mice, and the improvement was mainly attributed to the reconstructed protein components after extrusion (Yao & Ren, 2014). α‐Glucosidase, a key enzyme for carbohydrate digestion, has been considered as a therapeutic target for the modulation of postprandial hyperglycemia (Krentz & Bailey, 2005). α‐Glucosidase inhibitor, such as acarbose, has been widely used as a clinical drug to control postprandial blood glucose (Kim et al., 2014). In 2017, the International Diabetes Federation estimated that around 424.9 million people were involved in diabetes mellitus (IDF, 2017). In recent years, food‐based treatment for blood glucose control has been welcomed by more and more patients.

The aim of this work was to investigate the effect of extruded adzuki bean flour on wheat flour and steamed bread quality, including the farinograph and pasting characteristics of blended flours, steamed bread color parameters, textural properties, and sensory evaluation. Amino acid profile and α‐glucosidase inhibitory activity of CSB were also evaluated. The study could provide scientific evidence for the development of nutritional and biological steamed bread.

## 2 MATERIALS AND METHODS

### 2.1 Materials

Wheat flour (WF, High gluten, Beijing Yihai Kerry Grain, Oil and Food Industrial Co., Ltd) and adzuki bean were obtained from the local market of Beijing. Adzuki bean were ground in a laboratory mill and passed through an 80 mesh sieve. Extruded adzuki bean flour (EABF) was prepared by a twin laboratory screw extruder typed DS56 (Saixin machinery) according to our previous method (Yao & Ren, 2014). WF was partially substituted by EABF with a ratio of 10%, 20%, 30%, and 40% to gain different flour blends, respectively. Rat intestinal acetone powder was purchased from Sigma‐Aldrich. All other analytical chemicals used were purchased from Beijing Chemical Reagent.

### 2.2 Nutritional components analysis of flour samples

The contents of moisture, protein, starch, ash, and fat were determined according to AACC International Approved Methods of Analysis (11th Edition), Methods 44‐15, 46‐11, 76‐13, 08‐01, and 30‐10, respectively.

### 2.3 Farinograph tests of flour samples

Farinograph characteristics of flours were conducted by a Brabender Farinograph^®^‐E according to the AACC‐54‐21 method. The water absorption (WA), development time (DT), stability time (ST), farinograph quality number (FQN), and softening degree (SD) of the flour samples were obtained.

### 2.4 Pasting properties test of flour samples

The pasting properties of flours were performed in a Brabender Viscograph‐E as reference to the previous studies with slight modification (Lim & Seib, 1993; Rosell, Rojas, & Barber, 2001). Peak viscosity (PV), breakdown (BD), and setback (SB) were recorded.

### 2.5 Preparation of CSB

The CSB was performed according to previously published method (Huang, Betker, Quail, & Moss, 1993; Zhu, 2014). The recipe formula of CSB consists of 500 g WF or EABF blended flours, and 4 g dry yeast (Angle Yeast Co., Ltd), and the content of water was adjusted, respectively, on the basis of farinograph WA tests. WF Chinese steamed bread (WF‐CSB) and extruded adzuki bean–wheat composite flour Chinese steamed bread (EABF‐CSB) were gained accordingly.

### 2.6 Color measurements of CSB

The color test of steamed bread was performed by a High‐quality Spectrophotometer NS800 (3NH Technology Co., Ltd) and expressed as L^*^ (representing the lightness), a^*^(representing the variation from green to red), and b^*^ (representing the variation from blue to yellow) values. The measurements were performed in triplicates.

### 2.7 Texture profile analyses of CSB

CSB was cut into 25 mm slice in thickness from the center crumb and measured by a TA‐XT plus Texture Analyze (Stable Micro System) equipped with a probe (P/36R) (Sun, Jiang, Tian, Ling, & Lin, 2005). The average value of hardness, chewiness, recovery, springiness, and cohesiveness was showed after triplicate tests.

### 2.8 Specific volume of CSB

The specific volume (SV) of CSB was measured by the millet replacement method described by Luo et al. (2017). SV was calculated as the ratio between the volume and the weight (ml/g).

### 2.9 Sensory evaluation of CSB

Sensory evaluation of CSB was conducted by 9 panelists in triplicate according to Zhu (2014) and Chinese National Standards GB/T 35991‐2018 (Table 1) with slight modification in the sensory assessment standard of appearance and elasticity. All panelists are students trained with CSB as part of the diet. Each personnel were given the evaluation forms with a list of line scales accordingly.

**Table 1:** Sensory assessment standard of CSB

Parameters	Scores	Sensory assessment standard
Appearance	15	12–15, skin smooth; 8–11, shrink, collapse, presenting bubbles or burn spots; 1–7, coarse skin, lumps, asymmetry shape
Color	10	8–10, shiny; 6–7, slight dark; 1–5, red heavier
Structure	20	16–20, longitudinal section with small pores evenly; 11–15, uniform pores, but too fine or rough; 1–10, uneven pores or rough structure
Elasticity	20	16–20, great recovery character after finger press, great bite; 11–15, moderate; 1–10, lower recovery and bite
Tooth viscosity	10	8–10, great chewing without teeth stickness; 6–7, moderate; 1–5, chewing is not refreshing, sticky
Taste	5	4–5, WF or EABF fragrance, no smell; 3, moderate; 1–2, unpleasant smell
Total score	80	

### 2.10 Protein and amino acids content, and α‐glucosidase inhibitory activity assay of CSB and EABF

CSB was ground in a laboratory mill after freeze‐drying. The contents of protein and amino acids were carried out according to the Chinese National Standards GB 5009.5‐2016 and GB 5009.124‐2016, respectively.

The protein isolates of flour and steamed bread were prepared, and α‐glucosidase inhibitory activity of them (10 mg/ml) was determined according to the method as previously described by Yao and Ren (2014).

### 2.11 Statistical analysis

All values were expressed as mean ± standard deviation. Data were analyzed using one‐way analysis of variance followed by the post hoc LSD test on SPSS Statistical Software (SPSS version 22.0, IBM SPSS).

## 3 RESULTS AND DISCUSSIONS

### 3.1 Nutritional composition of flour samples

The contents of nutritional compositions in WF, ABF, and EABF were presented in Table 2. The content of protein and starch of ABF and EABF was consistent with previous study of adzuki bean reported by Tjahjadi and Breene (1984). Comparing to ABF, EABF showed a relative lower moisture and fat content, but nonsignificant higher protein, starch, and ash content. It seemed that extrusion showed no significant effect on the nutritional quality of ABF. The protein content and ash content of ABF and EABF were significantly higher than that of WF, while the content of starch and fat was lower than that of WF. It indicated that addition of EABF to steamed bread could provide more protein. Regarding ash content, it could be explained by the fact that adzuki bean was rich in mineral elements (Durak, Baraniak, Jakubczyk, & Świeca, 2013).

**Table 2:** Nutritional components of flour samples

Sample	Moisture (g/100 g)	Protein (g/100 g DW)	Starch (g/100 g DW)	Ash (g/100 g DW)	Fat (g/100 g DW)
WF	12.19 ± 0.03^a^	13.11 ± 0.43^b^	69.85 ± 0.27^a^	0.59 ± 0.02^b^	0.96 ± 0.03^a^
ABF	12.11 ± 0.10^a^	23.71 ± 0.56^a^	55.73 ± 0.63^b^	3.10 ± 0.03^a^	0.31 ± 0.06^b^
EABF	9.92 ± 0.05^b^	24.16 ± 0.38^a^	56.32 ± 1.31^b^	3.24 ± 0.03^a^	0.14 ± 0.02^c^

Note

Values are means ± standard deviation. Means with different letters in the same column are significantly (*p* < .05) different. Protein, starch, ash, and fat are expressed in dry weight (DW).

Abbreviations: EABF, extruded adzuki bean flour; WF, wheat flour.

### 3.2 Effect of EABF on farinograph parameters

The farinograph test results of WF and EABF blended flours were shown in Table 3. WA was increased by 4.90%–21.13%, when WF was partially substituted by EABF from 10% to 40%. This might be due to the increased content of protein in blended flour. WA depends on the protein and presence of damaged starch granules in the WF (Guttieri, Bowen, Gannon, O'Brien, & Souza, 2001). Protein exhibited high ability to compete for water with other constituents in the dough system and induced the increasing WA values (Doxastakis, Zafiriadis, Irakli, Marlani, & Tananaki, 2002; Martínez, Oliete, & Gómez, 2013). Furthermore, extrusion might induce damage to starch granules and lead to increase the contact surface between EABF starch particle and water (Rosell, Santos, & Collar, 2010); therefore, WA of the EABF blended flours was enhanced.

**Table 3:** Farinograph parameters of wheat flour and blended flours

Sample	WA (%)	DT (min)	ST (min)	FQN	SD (FU)
WF	68.50 ± 0.43^e^	8.05 ± 0.12^a^	9.68 ± 0.27^a^	118.38 ± 4.69^a^	29.67 ± 1.26^d^
10% EABF	73.40 ± 0.16^d^	6.93 ± 0.19^b^	8.20 ± 0.22^b^	106.98 ± 4.05^b^	75.91 ± 3.11^c^
20% EABF	78.40 ± 0.20^c^	5.61 ± 0.05^c^	6.47 ± 0.10^c^	79.66 ± 3.50^c^	93.25 ± 5.29^b^
30% EABF	82.83 ± 0.36^b^	2.23 ± 0.24^d^	3.49 ± 0.05^d^	35.90 ± 1.94^d^	97.98 ± 2.50^b^
40% EABF	89.63 ± 0.21^a^	1.01 ± 0.15^e^	1.39 ± 0.05^e^	22.92 ± 1.05^e^	120.52 ± 4.50^a^

Note

Values are means ± standard deviation. Means with different letters in the same column are significantly different (*p* < .05).

Abbreviations: DT, development time; EABF, extruded adzuki bean flour; FQN, farinograph quality number; SD, softening degree; ST, stability time; WA, water absorption; WF, wheat flour.

The DT, ST, and FQN of blended flours decreased with the addition of EABF. The previous study also showed that the addition of potato in WF could decrease the DT and ST value (Liu, Mu, et al., 2016). It has been reported that farinograph stability is mainly affected by the protein network (Konopka, Fornal, Abramczyk, Rothkaehl, & Rotkiewicz, 2004). Fu, Tian, Sun, and Li (2008) found that addition of nonwheat flour ingredients to WF could dilute gluten content, weaken the cross‐links between proteins, and reduce the interactions between the chains. The results in our study may be related to the dilution of WF gluten by EABF protein and then induced the degradation of the gluten matrix.

The SD of dough increased with increasing of EABF partially substitution with WF. The increase might be due to the reduced gluten content and increased WA of blended flours. It suggested that EABF could not enhance the gluten strength and thus weaken the ability of resistance to mixing capacity and farinograph quality of dough. Mohammed, Ahmed, and Senge (2012) also reported that addition of chickpea flour to WF induced the weakening of dough strength.

### 3.3 Pasting property of flour samples

Gelatinization of starch in WF, as a necessary part of cooking products, has a significant impact on wheat food quality (Batey, Curtin, & Moore, 1997). The pasting properties of starch can provide relevant information for structural characterization and food processing (Park, Chung, & Yoo, 2004). Table 4 showed the results of pasting parameters on WF and blended flours. It is clearly evident that all viscosity indexes decreased significantly with the substitution of EABF. The decline of PV might be due to the lower starch content of EABF than that of WF. To a large extent, the evaluation of pasting property was determined by the starch in the flours (Brennan et al., 2006). Teng, Liu, Bai, and Liang (2015) also observed the decrease in PV value when the starch content of WF was lowered by mixing with rice bran.

**Table 4:** Pasting property of wheat flour and blended flours

Sample	PV (BU)	BD (BU)	SB (BU)
WF	591.67 ± 1.89^a^	174.33 ± 2.87^a^	287.67 ± 4.19^a^
10% EABF	421.00 ± 5.89^b^	132.10 ± 3.40^b^	261.77 ± 6.84^b^
20% EABF	330.00 ± 4.97^c^	102.33 ± 1.25^c^	232.00 ± 5.10^c^
30% EABF	210.12 ± 4.03^d^	85.38 ± 0.47^d^	190.35 ± 5.66^d^
40% EABF	167.88 ± 2.36^e^	72.55 ± 0.47^d^	168.21 ± 2.05^d^

Note

Values are means ± standard deviation. Means with different letters in the same column are significantly different (*p* < .05).

Abbreviations: BD, breakdown; EABF, extruded adzuki bean flour; PV, peak viscosity; SB, setback; WF, wheat flour.

BD could be explained as the stability of starch gel, and it suggests the ability of shear and rupturing of swollen starch granules (Fu et al., 2008). BD values of the flours blended with EABF were significantly lower than that of WF. Tjahjadi and Breene (1984) reported that adzuki bean starch exhibited a higher resistance to swelling and rupturing than that of wheat starch. It indicated that EABF also presented similar pasting property. Wang, Opassathavorn, and Zhu (2015) found that addition of quinoa flour could lower the BD and enhance the ability to withstand heating and shear stress of WF. Therefore, the lower value of BD may indicate a better stability of blended flour.

SB could be used to evaluate the gelling ability of antiaging (Zaidul, Yamauchi, Kim, Hashimoto, & Noda, 2007). The decrease in SB caused by EABF addition might be mainly due to the increased WA of mixed flour, which would consequently induce limited water availability and inadequate starch gelatinization. It might be due to the high protein content of EABF hindered the rearrangement of the starch hydrogen bonds; therefore, the antiaging ability of the starch in blended flour was improved.

### 3.4 Color parameters of CSB

**Figure 1:**
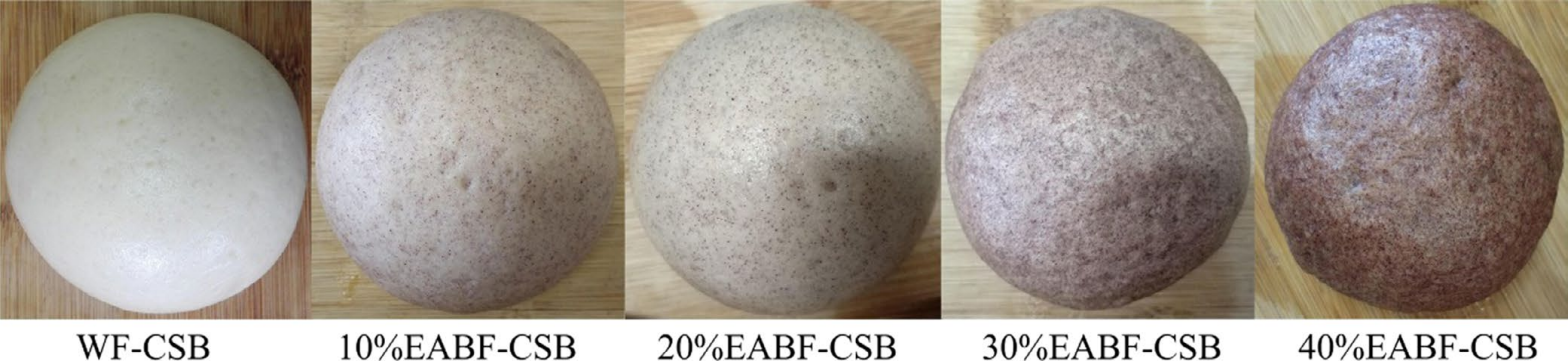
Photographs of the CSBs

Favorable color played a critical role in the attractive appearance of foods, and its change can be treated as an index of CSB (Zhu, Cai, & Corke, 2010). Therefore, it is necessary to quantitatively evaluate the CSB color. For CSB, addition of EABF decreased the value of L^*^and b^*^, while improved the value of a^*^(Table 5). There were significant differences between color values of WF‐CSB and EABF‐CSBs. As shown in Figure 1, the replacement of WF by EABF induced a gradually increased red color for steamed breads.

**Table 5:** Color parameters of CSBs with different levels of EABF addition

Sample	L^*^	a^*^	b^*^
WF‐CSB	75.81 ± 0.21^a^	0.69 ± 0.01^e^	14.96 ± 0.31^a^
10% EABF‐CSB	70.57 ± 0.37^b^	1.95 ± 0.02^d^	10.52 ± 0.26^b^
20% EABF‐CSB	67.98 ± 0.11^c^	2.57 ± 0.01^c^	10.26 ± 0.30^b^
30% EABF‐CSB	63.57 ± 0.43^d^	3.58 ± 0.01^b^	10.43 ± 0.21^b^
40% EABF‐CSB	63.36 ± 0.25^d^	3.96 ± 0.05^a^	9.90 ± 0.18^c^

Note

Values are means ± standard deviation. Means with different letters in the same column are significantly different (*p* < .05).

Abbreviations: EABF‐CSB, extruded adzuki bean flour Chinese steamed bread; WF‐CSB, wheat flour Chinese steamed bread.

### 3.5 Textural properties of CSB

The effect of EABF on textural characteristics of CSB was presented in Table 6. It can be observed that EABF has substantial influence on hardness and chewiness. Hardness was used as the index of the total textural attributes (Ulziijargal, Yang, Lin, Chen, & Mau, 2013). In this study, hardness was significantly increased with the increase in EABF addition. The higher degree of hardness may be caused by lower SV (Luo et al., 2018). It was likely that crumb hardening was involved with water migration from crumb to crust, hydrophilic components of flour, and starch retrogradation, as well as the interaction between gluten, starch, and water (Liu, Luo, Chen, Xu, & Liu, 2016). This led to the increased amount of solid components and a denser structure in CSB. Chewiness was also increased with the addition of EABF. This could be mainly attributed to the hardening of CSB (Luo et al., 2017).

**Table 6:** Texture characteristics of CSB

Sample	Hardness (g)	Chewiness	Recovery	Springiness	Cohesiveness
WF‐CSB	1,017.60 ± 58.05^d^	1,207.06 ± 76.94^d^	0.62 ± 0.01^a^	0.94 ± 0.01^a^	0.90 ± 0.02^a^
10% EABF‐CSB	1,306.46 ± 38.14^c^	1,561.61 ± 46.63^c^	0.61 ± 0.01^a^	0.93 ± 0.01^a^	0.90 ± 0.01^a^
20% EABF‐CSB	1,615.57 ± 51.32^b^	1,970.88 ± 84.80^b^	0.61 ± 0.01^a^	0.92 ± 0.02^a^	0.89 ± 0.01^a^
30% EABF‐CSB	1,714.20 ± 102.84^b^	2,114.00 ± 110.44^b^	0.58 ± 0.01^b^	0.92 ± 0.01^a^	0.88 ± 0.02^a^
40% EABF‐CSB	2,885.99 ± 107.15^a^	3,829.48 ± 161.52^a^	0.56 ± 0.01^c^	0.89 ± 0.02^a^	0.87 ± 0.01^a^

Note

Values are means ± standard deviation. Means with different letters in the same column are significantly different (*p* < .05).

Abbreviations: EABF‐CSB, extruded adzuki bean flour Chinese steamed bread; WF‐CSB, wheat flour Chinese steamed bread.

Recovery, springiness and cohesiveness values decreased with the increase in EABF addition. Lower value of CSB recovery means that it is easily damaged and deformed (Luo et al., 2017). The recovery value of 30% EABF‐CSB and 40% EABF‐CSB was significantly (*p* < .05) lower than that of WF‐CSB. Liu, Mu, et al. (2016) also found that addition amount of potato flour beyond 20% could significantly decrease the resilience of steamed bread. Springiness is the degree of recovery after being compressed at the first time. Cohesiveness reflects the internal cohesion of CSB, and lower cohesiveness indicates the susceptibility to fracture (Luo et al., 2017). Different from hardness, springiness value and cohesiveness value of CSBs were not significantly affected by EABF addition, although they were decreased. In previous reports about CSB with addition of calamondin fiber, the change of springiness and cohesiveness was also found not completely consistent with hardness (Fu, Shiau, & Chang, 2014). It seemed that hardness and chewiness were more sensitive to the addition of EABF than the other parameters of CSB. All these results indicated that excessive addition of EABF would induce negative effects on the texture of steamed bread.

### 3.6 Specific volume of CSB

**Figure 2:**
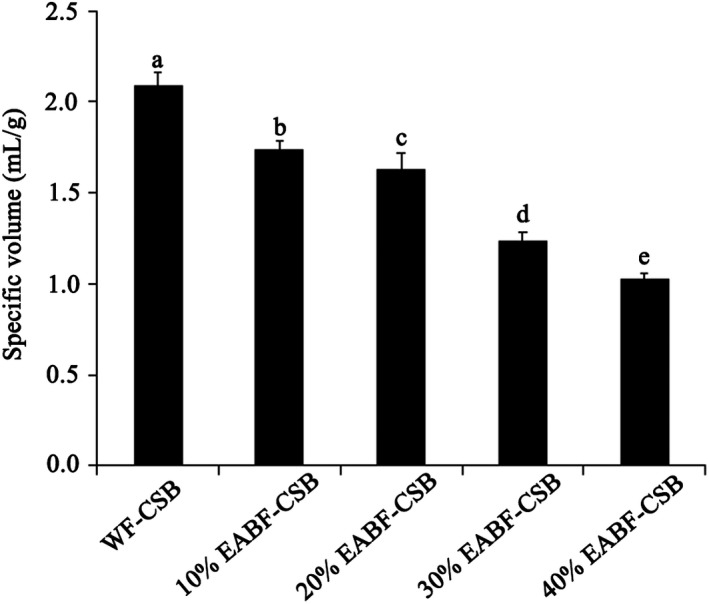
Specific volume of CSBs

The SV, mainly depended on the formation and expansion of gluten network, is the principal parameter to characterize volume expansion and gas‐holding capacity of dough during fermentation. Gluten protein plays a key role in SV (Liu, Luo, et al., 2016). Too large SV will result in a very open grain structure, whereas small SV will give a compact and closed dough structure (Sharadanant & Khan, 2003). SV of EABF‐CSBs was significantly lower than that of WF‐CSB, as could be seen in Figure 2. It is supposed that the diminished SV was due to the dilution of gluten (Luo et al., 2018).

### 3.7 Sensory evaluation of CSB

**Figure 3:**
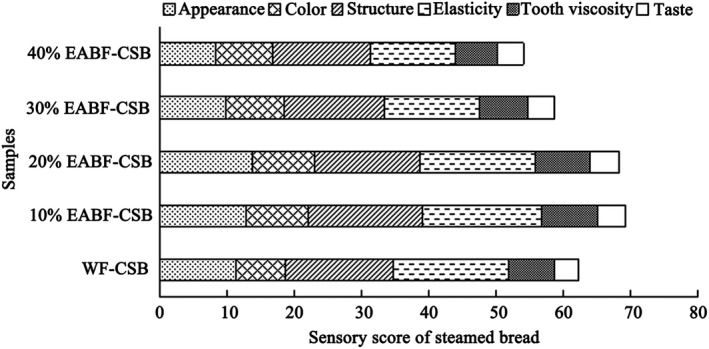
Sensory scores of CSB

It is worthy to evaluate the acceptability of CSB after addition of EABF, since the addition has induced the above changes of farinograph, pasting, and textural properties. Figure 3 showed the sensory evaluation of CSB samples. The scores of appearance and color rise first and then decline with EABF addition. It is likely that a majority of panelists prefer foods with altered smooth appearance and light red color with EABF (Figure 1). Therefore, they gave reasonable scores for 10% EABF‐CSB and 20% EABF‐CSB. Koletta, Irakli, Papageorgiou, and Skendi (2014) also found that color showed positive effect on the consumer acceptance for whole wheat bread. However, excessive addition (40%) of EABF would induce the partial surface collapse and dull the red color of steamed bread (Figure 1). It resulted in the relative low score of appearance and color.

The changes of structure, elasticity, and tooth viscosity of CSBs were observed when adding EABF. The highest score of these parameters were all found for 10% EABF‐CSB. The score of them was then decreased with the EABF addition from 20% to 40%. Interestingly, most panelists supposed that the puffed beany flavor of CSB with EABF addition was acceptable and favorable. It is notable that the flavor improved with EABF substitution from the increased scores of taste.

The total score of steamed bread with the 10% and 20% addition of EABF was significantly higher than that of WF‐CSB, while the score of 30% and 40% EABF‐CSBs was extremely lowered. This might be due to that excessive addition of EABF negatively affected the taste, appearance, and structure of CSB. Therefore, it can be concluded that the maximum addition of EABF is 20% in order to improve the sensory quality of steamed bread.

### 3.8 Protein and essential amino acids contents of CSB

Based on the sensory evaluation results, twenty percent was considered to be the upper limit amount of EABF addition. Therefore, the chemical and biological analyses of CSBs were only performed for the WF‐CSB, 10% EABF‐CSB, and 20% EABF‐CSB.

The protein and amino acid improvement of steamed bread could be benefited from addition of EABF (Table 7). EABF‐CSBs showed a better profile of protein and essential amino acid contents than that of WF‐CSB. All the protein and eight essential amino acid contents were improved in the CSB with addition of EABF. Compared with WF‐CSB, lysine, threonine, valine, isoleucine, leucine, and phenylalanine contents of EABF‐CSB at level 10% and 20% were significantly increased. It is notable that wheat was deficient in lysine and threonine, and correspondingly, WF‐CSB showed the same profile, especially for lysine. The lysine content in steamed bread was significantly increased by 104.35% and 152.17%, respectively, with addition of 10% and 20% EABF. This improvement could be attributed to the high lysine content of adzuki bean (Yao et al., 2011).

**Table 7:** Protein and essential amino acids content of CSB

Nutritional composition (g/100 g)	WF‐CSB	10% EABF‐CSB	20% EABF‐CSB
Protein	13.42 ± 0.28^c^	14.45 ± 0.14^b^	15.16 ± 0.14^a^
Lysine	0.23 ± 0.01^c^	0.47 ± 0.02^b^	0.58 ± 0.05^a^
Threonine	0.31 ± 0.01^b^	0.38 ± 0.03^a^	0.42 ± 0.02^a^
Valine	0.57 ± 0.02^c^	0.64 ± 0.03^b^	0.73 ± 0.04^a^
Isoleucine	0.48 ± 0.02^c^	0.59 ± 0.04^b^	0.68 ± 0.05^a^
Leucine	0.83 ± 0.07^c^	0.97 ± 0.09^b^	1.13 ± 0.10^a^
Phenylalanine	0.68 ± 0.03^b^	0.79 ± 0.04^a^	0.84 ± 0.05^a^
Methionine	0.14 ± 0.01^a^	0.16 ± 0.01^a^	0.16 ± 0.01^a^
Tryptophan	0.10 ± 0.01^a^	0.11 ± 0.01^a^	0.12 ± 0.01^a^

Note

Values are means ± standard deviation. Means with different letters in the same row are significantly different (*p* < .05). Values of protein and essential acids are expressed as g/100 g of wet weight.

Abbreviations: EABF‐CSB, extruded adzuki bean flour Chinese steamed bread; WF‐CSB, wheat flour Chinese steamed bread.

### 3.9 α‐Glucosidase inhibitory activity of CSB and flour

The α‐glucosidase inhibitory activity results of protein extracts from flour and steamed bread were shown in Table 8. After extrusion, the inhibitory activity of protein extracts in adzuki bean was significantly improved. Yao, Cheng, and Ren (2014) demonstrated that the restructured protein in EABF induced by extrusion was responsible for the improvement of α‐glucosidase inhibitory activity of adzuki bean. Furthermore, oral intake of extruded adzuki bean protein (300 mg/kg) was found to be effectively reducing the postprandial blood glucose of streptozocin‐treated rats (Yao et al., 2014). The previously reported α‐glucosidase inhibition rate of EABF (60.44%) was testified in this study (58.09%). Our results further indicated that the α‐glucosidase inhibitory activity of EABF protein could be maintained after steaming for CSB. It was apparent that there was a positive effect of EABF on the α‐glucosidase inhibitory activity of steamed bread. Compared with WF‐CSB, the α‐glucosidase inhibition rate of protein extracts from EABF‐CSBs was increased by 26.26% and 33.98%, respectively, with addition of 10% and 20% EABF. It seemed that CSB added with EABF showed a more nutritional and biological characteristics.

**Table 8:** α‐Glucosidase inhibitory activity of protein extracts from CSBs and EABF

Sample	α‐Glucosidase inhibition rate (%)
WF‐CSB	5.90 ± 0.12^e^
10% EABF‐CSB	32.16 ± 1.37^c^
20% EABF‐CSB	39.88 ± 1.94^b^
EABF	58.09 ± 2.72^a^
ABF	12.47 ± 1.05^d^

Note

Values are means ± standard deviation. Means with different letters in the same column are significantly different (*p* < .05).

Abbreviations: ABF, adzuki bean flour; ABF‐CSB, extruded adzuki bean flour Chinese steamed bread; EABF, extruded adzuki bean flour; WF‐CSB, wheat flour Chinese steamed bread.

## 4 CONCLUSION

Results showed that significant differences in nutritional composition between EABF and WF were characterized by higher protein and ash contents, and lower starch and fat. Compared with WF, the values of farinograph parameters (DT, ST, and FQN) and pasting properties with EABF blended flours were decreased. The sensory evaluation total score of CSB with EABF addition 10%–20% was improved, while accompanied by slight undesirable changes at SV and hardness. The addition amount of EABF above 30% would lead to strong red color, low SV, and undesirable eating quality of CSB. The protein and essential amino acid contents of 10% EABF‐CSB and 20% EABF‐CSB were significantly increased, especially for lysine. Furthermore, the two kinds of steamed breads showed higher α‐glucosidase inhibitory activity than that of WF‐CSB. It might be concluded that steamed bread with addition of 10% and 20% EABF could be successfully used to prepare steamed bread enriched in nutrition and biological activity.

## CONFLICT OF INTEREST

The authors declare that they have no conflict of interest.

## ETHICAL APPROVAL

The study has nothing to do with human or animal testing.
